# Correction to *Lancet Diabetes Endocrinol* 2015; 3: 243–53

**DOI:** 10.1016/S2213-8587(15)00145-X

**Published:** 2015-06

T*he Interleukin 1 Genetics Consortium Cardiometabolic effects of genetic upregulation of the interleukin 1 receptor antagonist: a Mendelian randomisation analysis.* Lancet Diabetes Endocrinol *2015; **3:** 243–53*—The open access licence was listed as “CC-BY-NC-ND” whereas it should have been “CC-BY”. This correction has been made as of May 21, 2015.

